# Effectiveness of simplifying antiretroviral therapy to maintain viral suppression and improve bone and renal health: comparing simplified and non-simplified therapy

**DOI:** 10.1016/j.bjid.2025.104578

**Published:** 2025-09-20

**Authors:** Juliana Olsen Rodrigues, Alexandre Naime Barbosa, Stephanie Valentini Ferreira Proença, Lenice Rosário de Souza

**Affiliations:** Universidade Estadual Paulista (UNESP), Departamento de Infectologia, Botucatu, SP, Brazil

**Keywords:** HIV, tenofovir, simplification, osteopenia, osteoporosis, glomerular filtration rate

## Abstract

**Objective:**

Nucleoside/nucleotide reverse transcriptase inhibitors, particularly tenofovir, can cause long-term side effects such as decreased bone mineral density and estimated glomerular filtration rate. A strategy to mitigate these effects is the simplification of antiretroviral therapy, which involves withdrawing one of the nucleoside/nucleotide reverse transcriptase inhibitors from the therapeutic scheme. While clinical trials and real-world studies have demonstrated that the simplified therapy maintains undetectable viral loads, its impact on bone mineral density and kidney function remains unclear owing to the lack of real-world evidence.

**Methods:**

This retrospective cohort study compared 152 patients who underwent antiretroviral therapy simplification (primarily due to osteopenia, osteoporosis, or decreased estimated glomerular filtration rate) with 306 patients who maintained triple therapy, between April 2013 and September 2022. The simplified regimens included lamivudine plus dolutegravir or ritonavir-boosted darunavir. The groups were analyzed based on their demographic characteristics using Student's *t-*test in the case of symmetric data. Therapeutic success (undetectable viral load at the end of follow-up) was assessed using Kaplan Meier survival analysis. The estimated glomerular filtration rate variation before and after simplification was analyzed using the Mann-Whitney test. Pre-and post-simplification bone mineral density values were evaluated using the chi-square test for trends and assessed in the simplified therapy group. A significance level of 5% (α = 0.05) was adopted for all tests.

**Results:**

Simplified antiretroviral therapy was non-inferior to triple therapy in maintaining undetectable viral load. Patients receiving simplified regimens showed a positive variation in estimated glomerular filtration rate. A small subset of patients also exhibited improvements in bone mineral density after antiretroviral therapy simplification.

**Conclusions:**

These findings suggest that simplified therapy is as effective as triple therapy and has the additional benefit of reducing tenofovir-related adverse events.

## Introduction

In June 1981, the first cases of Acquired Immunodeficiency Syndrome (AIDS) were described by the Centers for Disease Control and Prevention, in men who have sex with men, in New York and Los Angeles, USA. This was the beginning of the Human Immunodeficiency Virus (HIV)/AIDS pandemic, which has resulted in more than 84 million infections and 40 million deaths worldwide in the last 40-years.^1^^,^^2^ At the beginning of the pandemic, illness and death from HIV/AIDS were almost inevitable within approximately 8 to 10 years after infection because the drugs available at the time were incapable of achieving sustained virological suppression.^2^ Highly Active Antiretroviral Therapy (HAART), introduced in 1996, containing three active drugs from at least two different classes, significantly reduced the risk of developing AIDS and the number of deaths from the disease. In the last two decades, HAART has been able to maintain viral suppression in the long term, and as a result, the life expectancy of People Living with HIV (PLHIV) is currently very similar to that of the general population.^1^, ^2^, ^3^

Healthy aging has become a major goal in the treatment of PLHIV. Complications and comorbidities not associated with the virus, such as cardiovascular, bone, and kidney diseases, are currently more important causes of morbidity in PLHIV than opportunistic infections.^4^ Tenofovir (TDF) is one of the drugs of choice for starting HAART and is associated with nephrotoxicity and decreased Bone Mineral Density (BMD).^5^ Nephrotoxicity is related to an accelerated decline in Glomerular Filtration Rate (GFR) and proximal tubular dysfunction.^6^, ^7^, ^8^ Estimated Glomerular Filtration Rate (eGFR) should be measured every six months in PLHIV who are stable using the Chronic Kidney Disease Epidemiology Collaboration (CKD-EPI) formula, which is based on serum creatinine levels. Current guidelines for the treatment of PLHIV recommend screening for osteoporosis or osteopenia in postmenopausal women and men over 40-years of age.^9^ Some studies have suggested that PLHIV may be at risk of osteopenia/osteoporosis if they have been exposed to TDF for more than five years.^10^^,^^11^

Given the need to maintain treatment for life to prevent disease progression and reduce the risk of morbidity and mortality, simplified Antiretroviral Therapy (ART) options are being studied to minimize toxicity and maintain treatment effectiveness. Current HIV treatment guidelines recommend regimens consisting of two nucleoside/Nucleotide analog Reverse Transcriptase Inhibitors (NRTIs), as the backbone, combined with a third agent that may comprise a Non-Nucleoside analog Reverse Transcriptase Inhibitor (NNRTI), a protease inhibitor boosted with ritonavir (PI/r), or an integrase inhibitor.^1^, ^2^, ^3^

Strategies for simplification include therapies with two classes of antiretrovirals that have been proven effective in over 95 % of patients in several Randomized Clinical Trials (RCTs) demonstrating the efficacy of dual therapy: PI/*r* + lamivudine (3TC)^7^ and 3TC + Dolutegravir (DTG).^8^

Real-life observational studies remain scarce; however, they are emerging in the same direction, proving the effectiveness of simplified schemes based on PI/*r* + + 3TC^9^^,^^10^ and DTG + 3TC.^11^^,^^12^ In these studies, virological failure rates were less than 10.0 % (similar to that in RCTs) in analysis periods ranging from two to three years.

Before simplification, a review of the patient’s ART history and previous virological failures should be carried out by analyzing the presence of resistance mutations in genotyping tests, drug interactions, and the patient’s desire to change therapy, because adherence must be excellent in these cases. Furthermore, the main antiretroviral agent in the simplified therapy must have high potency and genetic barrier.^11^

In Brazil, 3TC combined with DTG or Darunavir boosted with Ritonavir (DRV/r) were evaluated for ART simplification strategies, as they have a high genetic barrier and demonstrate safety and efficacy in maintaining virological suppression.^12^

Most patients who are candidates for simplification already have an injury related to the long-term use of TDF, such as osteopenia/osteoporosis or decreased renal function. Assuming that withdrawing TDF from the regimen would improve these lesions, simplification would be an optimal treatment strategy because other NRTI options for maintaining triple therapy may have serious side effects.

The objectives of this study were to demonstrate the non-inferiority of simplified ART with 3TC + DTG and 3TC + DRV/r in maintaining undetectable VL compared to triple therapy, and to determine whether simplification of the scheme improves glomerular filtration rate and BMD.

## Materials and methods

This study was conducted at the Specialized Ambulatory Service of Infectology, located in a city in the interior of São Paulo, Brazil. It was designed as a retrospective observational cohort study comparing two groups of PLHIV: those who underwent ART simplification and a non-randomized comparison group selected at a 1:2 ratio from the same service and period.

The simplified group consisted of 153 PLHIV who transitioned to a simplified regimen with lamivudine (3TC) combined with either Dolutegravir (DTG) or ritonavir-boosted Darunavir (DRV/r). The comparison group included 306 PLHIV who continued on a triple ART regimen, consisting of one of the following combinations: 2 NRTIs + 1 NNRTI, 2 NRTIs + 1 PI/r, or 2 NRTIs + 1 INI.

Eligible participants were aged 18 years or older and had an undetectable VL at baseline. Clinical, antiretroviral, demographic, and virological data from both groups were collected between April 2013 and September 2022 from electronic medical records. These records were accessed from March 2021 to October 2022 using patients' medical record numbers.

One patient whose regimen was simplified to DTG + DRV/r was excluded from the analysis. Consequently, the total number of participants in the simplified group was adjusted to 152.

As a non-interventional study, the decision to simplify the ART regimen for each participant was determined by attending physicians. The main criteria for simplifying ART were the presence of osteopenia or osteoporosis on dual-energy X-Ray Absorptiometry (DXA) and/or an eGFR < 75 mL/min/1.73 m^2^. Simplification was also performed in patients with side effects related to other NRTIs, such as anemia and lipodystrophy associated with AZT, increased cardiovascular risk with Abacavir (ABC) use, or discontinuation of didanosine (ddI), an NRTI that was withdrawn from the therapeutic arsenal for HIV in Brazil in 2016. The exclusion criteria were previous virological failure, presence of mutations in previous genotyping tests, and chronic hepatitis B virus infection.

The eGFR was calculated based on serum creatinine levels using the CKD-EPI equation^13^ in both groups at the beginning and end of follow-up. Renal function was categorized by CKD (Chronic Kidney Disease) stages according to the 2012 KDIGO (Kidney Disease: Improving Global Outcomes) classification: G1 (≥ 90), G2 (60–89), G3a (45–59), and G3b (30–44) mL/min/1.73 m^2^.^14^

The lumbar spine and femoral neck were evaluated using the DXA system, which is routinely performed at our facility for patients receiving combined therapy with TDF for more than five years, men over 40-years of age, and postmenopausal women, in accordance with current clinical guidelines. BMD was evaluated pre- and post-simplification in the simplified group only. Patients in the non-simplified group did not undergo BMD assessment, as they did not meet established criteria for routine DXA evaluation.

A total of 43 patients in the simplified group had BMD measured pre- and post-simplification.BMD results were classified based on T-scores, as follows: greater than −1.5 indicates normal BMD; between −1.5 and −2.5 indicates osteopenia; and less than −2.5 indicates osteoporosis. The results were interpreted according to the “Consensus of the Brazilian Society of Clinical Densitometry of 2008″.^15^

### Statistical analysis

Frequencies and percentages were computed for categorical variables. These analyses were stratified into two groups: simplified and non-simplified. Therapeutic success (undetectable VL at the end of follow-up) was assessed using Kaplan Meier survival analysis (log rank statistics).

Comparisons of means between groups for quantitative variables were performed using Student's *t-*test in the case of symmetric data. In the case of asymmetry, gamma distribution adjustments were used for comparisons. The eGFR variation before and after simplification was analyzed using the Mann-Whitney test. Changes in CKD stage before and after simplification were evaluated using the Stuart-Maxwell test for paired categorical data. Pre-and post-simplification BMD values were associated with the Chi-Square test for trend. A significant level of 5 % (α = 0.05) was adopted for all tests, or the corresponding p-value was considered. All analyses were conducted using SAS for Windows (version 9.4) and SPSS 27 (IBM, Armonk, NY, USA).

## Results

**Fig. 1:**
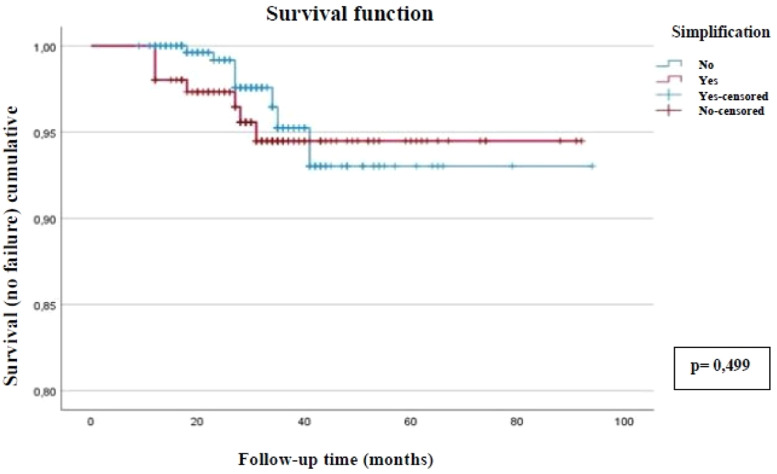
Kaplan-Meier curve illustrating therapeutic success (undetectable viral load) among 458 people living with HIV, comparing simplified and non-simplified antiretroviral regimens. Botucatu, Brazil, 2022.

No differences were observed between groups; among the 306 patients in the non-simplified group, 298 (97.4 %) remained with undetectable VL at the end of follow-up, and the same occurred in 148 (95.4 %) patients in the simplified group (*pp* = 0.499). According to Fig. 1, the permanence of patients with undetectable VL over time was similar between the two groups.

At the end of follow-up, seven patients in the simplified group and eight patients in the non-simplified group had detectable VLs. Poor adherence was the main reason, occurring in 4.6 % of simplified and 2.6 % of non-simplified patients, predominantly in younger males with psychosocial vulnerabilities.

The groups were heterogeneous in terms of the mean age (*p* < 0.001) and race (*p* = 0.0076). Regarding sex, the groups were homogeneous, as they had mostly male patients (*p* = 0.1972). Regarding the follow-up time, a difference was observed between the groups, as the simplified group had an average follow-up of 34-months, whereas the non-simplified treatment group was followed up for an average of 29-months (*p* < 0.001). The data are presented in Table 1.

**Table 1:** Demographic characteristics of the 458 people living with HIV (Botucatu, 2022).

Variables	Simplified (*n* = 152)	Non-simplified (*n* = 306)	p-value
Age (years)	54	42	<0.001
Sex, n ( %)			
Male	96 (63.2)	174 (56.9)	0.1972
Female	56 (36.8)	132 (43.1)	
Race, n ( %)			
White	134 (88.2)	255 (83.3)	
Brown	13 (8.6)	16 (5.2)	0.0076
Black	5 (3.3)	35 (11.4)	
Follow-up time (months)	34	29	<0.001

n, Number. Student *t*-test.

Among the 152 patients who underwent regimen simplification, 65 (42.7 %) transitioned to a regimen with 3TC + DRV/r, while 87 (57.2 %) transitioned to one with 3TC + DTG. The primary reasons for treatment simplification were osteopenia/osteoporosis, reduced eGFR, discontinuation of other NRTIs (ddI, AZT, or ABC), and other medical indications. The category “other medical indications” included cases involving drug interactions, comorbidities, or concurrent use of medications with a potential risk of causing renal or bone toxicity, as detailed in Table 2.

**Table 2:** Characteristics of the 152 individuals living with HIV in Group 1 (patients with simplified antiretroviral regimens), categorized by the reasons for simplification (Botucatu, 2022).

Reasons for simplification	Simplified patients (*n* = 152) n ( %)
Osteopenia/Osteoporosis	71 (46.7)
Decreased eGFR	50 (32.9)
Discontinuation of other NRTIs (ddI, AZT or ABC)	24 (15.8)
Other medical indications	7 (4.6)

Regarding the ART regimens before simplification, 46 patients (30.0 %) were using 3TC + TDF + NNRTI, 42 patients (27.4 %) were on 3TC + TDF + INI, and 40 patients (26.1 %) were receiving 3TC + TDF + IP/r. Additionally, 25 patients (16.3 %) were on regimens without TDF, consisting of 3TC combined with ABC, AZT, or ddI, along with IP/r or an NNRTI.

Among the 306 patients who maintained TDF, 169 (55.2 %) received 3TC + TDF + INI, 86 (28.1 %) received 3TC + TDF + IP/r, and 48 (15.6 %) received 3TC + TDF + NNRTI. Only three patients (0.9 %) were on regimens without TDF: ddI + 3TC + LPV/r, AZT + 3TC + DRV/r, and ABC + 3TC + DTG. Among these patients, a significant decrease in renal function (*p* < 0.001) was observed over a period of 29.4 ± 11.2 months.

**Fig. 2:**
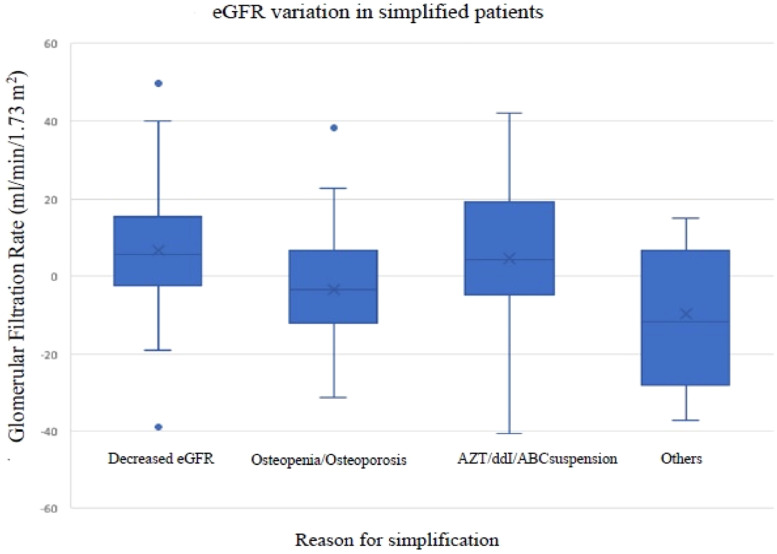
Variation in the estimated glomerular filtration rate among 152 people living with HIV on simplified antiretroviral regimens, categorized by the reason for regimen simplification. Botucatu, Brazil, 2022.

In the simplified ART group (*n* = 152), 50 patients had their antiretroviral regimen simplified due to reduced renal function. The variation in eGFR (calculated as final eGFR minus initial eGFR) in these patients demonstrated significant improvement when compared to the eGFR variation observed in patients who underwent regimen simplification for other reasons such as osteopenia/osteoporosis or other medical indications (*p* < 0.05, Mann-Whitney test, Fig. 2). Among these patients, the mean delta eGFR was +6.85 mL/min/1.73 m^2^ with a Standard Deviation (SD) of 15.0, over a mean follow-up period of 34-months.

Although eGFR variation improved among patients with renal dysfunction, when analyzing CKD classification before and after simplification, 33 patients (26 %) improved, 22 (17 %) worsened, and 96 (57 %) remained stable. However, no statistically significant shift in CKD staging distribution was observed (Stuart-Maxwell χ^2^ = 6.20; degrees of freedom [df = 6]; *p* = 0.40), suggesting overall stability in renal function categories following ART simplification.

In the non-simplified group (*n* = 306), who maintained TDF-based regimens, a significant decline in renal function was observed. The mean variation in eGFR over a follow-up period of 29-months was −4.7 mL/min/1.73 m^2^ (SD = 13.2).

In the group of 152 patients who underwent treatment simplification, 43 had osteoporosis or osteopenia as the primary indication for simplification. Among these 43 patients, 13 (72.2 %) showed improvement in BMD, with normalization of bone mass. Additionally, 22 patients (78.6 %) showed regression from osteoporosis to osteopenia (*p* < 0.0001). No patient experienced worsening of BMD during the follow-up period, which lasted an average of two and a half years after simplification.

## Discussion

This retrospective cohort study verified that simplified ART had the same effectiveness as triple therapy in relation to the maintenance of undetectable HIV VL, with no difference between the groups. This result is consistent with other real-life studies that used 3TC + DTG^16^, ^17^, ^18^, ^19^, ^20^ or 3TC + DRV/r^16,21^ as a strategy for simplifying ART and reducing side effects related to NRTIs, especially TDF.

Regarding patients who had a VL above the detection limit at the end of the follow-up period, the reasons were non-adherence and abandonment of therapy, as observed in most real-life studies, with no presence of resistance mutations.^16^, ^17^, ^18^, ^19^, ^20^, ^21^ The reasons for non-adherence and abandonment of therapy in the present study included psychological and social issues, and were not related to adverse events, according to data from medical records.

Unlike RCTs, our study groups were heterogeneous regarding baseline characteristics such as race, age, and follow-up time. The absence of randomization introduces selection bias, as regimen simplification was physician-directed rather than randomly assigned. This limitation emphasizes the potential impact on group comparability. Specifically, patients in the simplified group were older on average than those in the non-simplified group. This probably occurred because losses in renal function and bone mass usually worsen with advancing age and longer exposure to TDF,^22^, ^23^, ^24^ which were the main clinical indications for ART simplification in this cohort. Regarding race, although the difference between groups was statistically significant, this imbalance was not based on clinical criteria or patient selection. It may reflect demographic variation within our service population or random distribution in this real-life, non-randomized cohort.

One of the desired outcomes was an improvement in the renal function after simplification. Although the present study showed a statistically significant mean improvement in eGFR among patients who underwent ART simplification due to reduced renal function, the clinical significance was limited, as most patients remained within the same CKD stage. This indicates that while simplification may lead to improvements in renal function, these changes are not sufficient to alter CKD staging. Some RCTs have shown improvements in renal function 48-weeks after discontinuing TDF. .^25^^,^^26^ In these studies, renal function was evaluated using eGFR calculations based on cystatin C levels in addition to the analysis of proximal tubule injury markers and the urinary protein/creatinine ratio.^25^^,^^26^

In a prospective cohort study, Maggiolo et al.^27^ observed a significant increase in serum creatinine levels two months after simplification in 94 patients simplified to a 3TC + DTG regimen. However, this increase was limited and stabilized after six months. This change aligns with the known mechanism of DTG as an inhibitor of the renal organic cation transporter 2, which mediates tubular creatinine secretion.^27^

To better assess renal function following the discontinuation of TDF from the regimen, alternative parameters, such as serum cystatin C levels, proteinuria, urinary electrolytes, and urinary protein-to-creatinine ratio, may provide more accurate insights. Estimating creatinine clearance can be unreliable, as an increase in serum creatinine may reflect DTG-induced alterations in tubular secretion or increased muscle mass, rather than actual renal impairment, as suggested by Maggiolo et al.^27^ Cystatin C levels are not routinely assessed because of the high cost in Brazil; however, other urinary parameters can be assessed.

Although some studies have suggested eGFR improvement after discontinuing TDF, factors such as older age, longer duration of HIV infection, and comorbidities contributing to renal function decline have been associated with a reduced likelihood of complete renal recovery.^22^^,^^26^ In the present study, comorbidities among PLHIV, such as diabetes, hypertension, and dyslipidemia, were not assessed, nor were the duration of HIV infection before regimen simplification, or prior TDF exposure, representing additional limitations that may have influenced renal and bone outcomes. These aspects should be explored in future studies to provide a more comprehensive analysis.

Another outcome analyzed was BMD following regimen simplification, given that previous studies have reported improvements in BMD as early as 48-weeks after TDF discontinuation.^25^^,^^28^ In the reviewed randomized clinical trials, patients who transitioned to simplified regimens showed improvements in BMD parameters and bone resorption markers compared with those who continued TDF-based therapy.^25^^,^^28^ In the present study, BMD improvement was observed in 23.3 % of patients with baseline osteoporosis or osteopenia, while most patients maintained stable BMD values during the follow-up period, which averaged two and a half years post-simplification. Notably, no patient experienced BMD worsening ‒ an outcome that might have occurred had TDF been maintained. However, the absence of a non-simplified control group for BMD assessment in this study limits causal inference. Furthermore, increasing the sample size of patients with BMD measurements before and after simplification in our study could provide more robust evidence and allow for a more comprehensive characterization of the effects of ART simplification on bone mineral density.

An alternative method of evaluation involves the analysis of bone remodeling biomarkers such as bone alkaline phosphatase, serum osteocalcin, C-terminal telopeptide, and type 1 collagen propeptide. Evidence from two RCTs demonstrated that these markers reduced in regimens excluding TDF, indicating decreased bone loss.^25^^,^^28^ However, these biomarkers were not assessed in our study.

Despite some limitations, such as the demographic heterogeneity of the groups and the relatively small sample size, the results of the present study are robust and consistent with those of other real-life studies and RCTs. Furthermore, this was a pioneering study comparing two groups of patients (simplified and non-simplified) in an observational manner.

More real-life studies are needed to prove the effectiveness of simplified therapy in maintaining viral suppression and verify the benefits in relation to bone and renal health. In addition, further studies are needed to determine the best moment to perform ART simplification to avoid these changes.

## Ethical approval statement

This study was approved by the Research Ethics Committee of Botucatu Medical School – UNESP (State University of São Paulo) on July 5th, 2022, under the ethical approval reference number: 59,638,222.0.0000.5411.

## Funding

There was no funding source for this study.

## Conflicts of interest

The authors declare that they have no known competing financial interests or personal relationships that could have appeared to influence the work reported in this paper.
